# Severe protein deficiency induces hepatic expression and systemic level of FGF21 but inhibits its hypothalamic expression in growing rats

**DOI:** 10.1038/s41598-021-91274-4

**Published:** 2021-06-14

**Authors:** Joanna Moro, Catherine Chaumontet, Patrick C. Even, Anne Blais, Julien Piedcoq, Claire Gaudichon, Daniel Tomé, Dalila Azzout-Marniche

**Affiliations:** UMR PNCA, AgroParisTech, INRAe, Université Paris-Saclay, 16 rue Claude Bernard, 75005 Paris, France

**Keywords:** Biochemistry, Biological techniques, Physiology

## Abstract

To study, in young growing rats, the consequences of different levels of dietary protein deficiency on food intake, body weight, body composition, and energy balance and to assess the role of FGF21 in the adaptation to a low protein diet. Thirty-six weanling rats were fed diets containing 3%, 5%, 8%, 12%, 15% and 20% protein for three weeks. Body weight, food intake, energy expenditure and metabolic parameters were followed throughout this period. The very low-protein diets (3% and 5%) induced a large decrease in body weight gain and an increase in energy intake relative to body mass. No gain in fat mass was observed because energy expenditure increased in proportion to energy intake. As expected, *Fgf21* expression in the liver and plasma FGF21 increased with low-protein diets, but *Fgf21* expression in the hypothalamus decreased. Under low protein diets (3% and 5%), the increase in liver *Fgf21* and the decrease of *Fgf21* in the hypothalamus induced an increase in energy expenditure and the decrease in the satiety signal responsible for hyperphagia. Our results highlight that when dietary protein decreases below 8%, the liver detects the low protein diet and responds by activating synthesis and secretion of FGF21 in order to activate an endocrine signal that induces metabolic adaptation. The hypothalamus, in comparison, responds to protein deficiency when dietary protein decreases below 5%.

## Introduction

Protein intake below nutritional needs has adverse effects on the organism^1^, and protein intake is therefore tightly controlled^2^. In rats, the requirement for protein is 15% energy^3^, while the recommendation is 20% energy (American Institute of Nutrition (AIN)). Different studies have reported that low protein diets (5% and 6% energy) induce an increase in food intake to maintain an adequate level of protein intake^4–6^. On the one hand, this low protein diet-induced hyperphagia leads to an increase in energy intake with a risk of increased adiposity^6–8^. On the other, the increase in total energy expenditure could partially prevent a gain in fat mass induced by the increased food intake^9–12^.

The Fibroblast Growth Factor 21 (FGF21), a liver-derived circulating hormone (hepatokine) belonging to the Fibroblast Growth Factor (FGF) family, was shown to respond to different nutritional signals^13^. Glucose and fructose induce liver FGF21expression and secretion in vivo^14^, and in primary rat hepatocyte culture, high glucose concentration induces FGF21 expression^15^. In humans, lipid infusion and a ketogenic diet increase blood FGF21 levels^16,17^. Interestingly, FGF21 also responds to different nutritional deficiencies, such as those caused by low protein diets as well as amino acid and choline-deficient diets^18–21^. All of these diets have been observed to increase circulating FGF21 levels and hepatic expression of *Fgf21* in mice and rats^11,15,18,22–24^. The effects of a low protein diet on food intake, energy expenditure and body weight involve FGF21. This was demonstrated when protein restriction in Wild type mice, but not in FGF21-KO mice, induced an increase in food intake and energy expenditure and a decrease in body weight and adiposity^25^.

FGF21 is expressed mainly in the liver^13,26^, but also in the pancreas, skeletal muscle, the gastrointestinal tract, testes, brown adipose tissue (BAT), white adipose tissue and the hypothalamus^27–29^. It is a member of a subfamily of the FGF family that has an endocrine, autocrine and paracrine signal^30^. When released into circulation, FGF21 regulates various physiological processes, including glucose and lipid metabolism^31^ and body weight control and energy expenditure^32^. It is also described as a satiety signal involved in the preference for sugars^33^ and alcohol^34^. In these different effects, hepatic FGF21 plays a main regulatory role both locally and systemically after its release in the blood. Nonetheless, the autocrine, paracrine or endocrine role of FGF21 expressed and produced in other sites such as the hypothalamus remains unknown. To our knowledge, no study has investigated the impact of different levels of protein deficiency on the relative hepatic and systemic expression and variation of FGF21 and on hypothalamic expression.

The present study aims to assess the dietary regulation of hepatic and hypothalamic FGF21 and their contribution to the metabolic and behavioral adaptations to protein restriction. Young growing rats were fed diets differing in protein percentages: very low (3% and 5%), moderately low (8% and 12%), or adequate (15% and 20%). The impact on body weight, body composition, food intake and energy expenditure was measured.

## Material and methods

### Ethics approval

The exploratory study was approved by the Regional Animal Care and Ethical Committee and the Minister of Research and conformed to the European legislation on the use of laboratory animals (registration number: APAFIS#13436-2017122616504600) and the was carried out in compliance with the ARRIVE guidelines. Criteria used for including and excluding animals were reported in APAFIS. In this study, no animals was excluded.

### Animals

Thirty-six, 3-week-old male Wistar Han rats (HsdHan: WIST, Envigo, France), weighing 50–55 g on arrival, were housed in a light and temperature-controlled animal facility (12-h light/12-h dark cycle, lights on from 00:00 to 12:00, 22 ± 1 °C). After one week of adaptation, during which they were fed with a standard rat chow diet (Régime croquettes from Safe, 16.10% protein), they were housed in individual cages and switched to their experimental diet for three weeks. All rats had ad libitum access to food and water during the experimental period.

### Pellet preparation

Food pellets were prepared using powder manufactured at the “Atelier de préparation des aliments” (UPAE, INRA, Jouy en Josas, France). Water was added to form a dough in order to avoid scattering of the powder and to measure food intake. This dough was cut into individual portions and left to dry for three days before being given to the rats. Each day, the evaporation rate was calculated to adjust the dry weight ingested by rats.

### Experimental design

After one week of adaptation to laboratory conditions, the 36 rats were randomly divided into six groups (n = 6/group, using G Power calculation, with α = 0.05; 1 − β = 0.8; f = 0.5). Each group was assigned one of the six isocaloric diets containing 3% (P3), 5% (P5), 8% (P8), 12% (P12), 15% (P15) or 20% (P20) protein for three weeks (Supplementary Table 1). Throughout the course of the experiment, a calibrated meal of 4 g (58.2 kJ) was given every day at 12:00 (onset of the night period) in order to train the rats to rapidly ingest the meal. Then, ad libitum access to food was given between 12:30 and 9:00 the next day. Food intake and body weight were measured daily throughout the three weeks. During the third week, each rat was placed for 48 h in a cage connected to an indirect calorimeter (24 h of adaptation and 24 h of measurement) to measure energy expenditure and spontaneous motor activity.

At the end of the experimental period, the rats were fed with a calibrated meal (4 g (58.2 kJ) at 11:00) of their test diet and were anesthetized with Isoflurane two hours later. Blood samples were taken from the portal vein, then from the vena cava until death. Glycaemia was immediately measured using a blood glucose meter (Life-Scan, One touch vita). After centrifugation (4 °C, 3000 rpm, 10 min), plasma was collected, aliquoted and stored at − 80 °C until analysis. Body composition was analyzed by dissection and weighing of the main tissues and organs. Pieces of brown and white adipose tissues, muscle, liver and the hypothalamus were frozen in liquid nitrogen (− 80 °C) for further measurement of mRNA abundance and biochemical analysis.

### Measurement of total energy expenditure, basal metabolism and spontaneous motor activity

Throughout 24 h of measurement, rats were housed individually in ventilated cages placed on an activity platform equipped with force transducers that produced an electric signal proportional to the intensity of work produced by rat activity^35^. For gas analysis, four cages at a time were simultaneously ventilated at 500 ml/min and each connected in turn to the gas analyzers. Oxygen (O_2_) consumption and carbon dioxide (CO_2_) production were measured in each cage for two min every ten min (two min for each cage and two min of room air to correct values for room O_2_% and CO_2_%). The first day was used as a habituation day and the second day was used for data analysis. Total metabolic rate (TMR) was calculated with the Weir formula^36^, modified to obtain data in Joules/seconds (Watts). The activity signal was continuously recorded at 100 Hz, averaged, stored at two sec intervals and post-experimentally synchronized to the VO_2_ and VCO_2_ signals. Mean daily values for resting metabolic rate (RMR) were calculated as the origin of the correlations between TMR and Activity. 24 h resting (REE) and total (TEE) energy expenditure were computed in kJ by multiplying RMR and TMR by 86.4 (3600 * 24 / 1000). To take into account between-group differences of body weight and body composition, energy intake, TEE and REE were normalized to the metabolically active mass (MAM) (i.e. the weight of the lean body mass + 20% of weight of fat mass). This mode of correction calibrated the adjustment to lean body mass by taking into account that the metabolic activity of fat mass is about ~ 20% of that of lean body mass, rather than null^35,37^.

### Plasma assays and mRNA measurement

Plasma FGF21 was determined using an enzyme-linked immunoassay (Mouse and Rat FGF-21 ELISA, BioVendor). Plasma insulin was measured with a Luminex assay (RMHMAG-84 K, MILLIPLEX Rat Metabolic Magnetic Bead Panel, Merck-Millipore).

Total RNA was extracted from duodenal, liver, muscle, brown adipose tissue, epididymal adipose tissue and the hypothalamus using TRIzol reagent (Invitrogen). RNA concentration was measured using a nanodrop spectrophotometer at 260 nm, and RNA integrity was verified by electrophoresis on agarose gel. Retrotranscription was performed on 0.4 µg of RNA using the High Capacity cDNA Archive Kit (Applied Biosystems). To measure gene expression, real time PCR was performed using Power SYBR Green PCR Master Mix (Applied Biosystems) on the Step One (Applied Biosystems) with 5 ng of cDNA. Gene expression was calculated as 2^−ΔCT^, where ΔCT = CT_Gene_ − CT_18S_. In order to detect potential contamination, negative controls were used (control without RT or RNA). Primer Express was used to design the primer sequences of genes, and the sequences of primers used are described in Supplementary Table 2.

### Statistical analysis

Data are presented as means ± SEM. Statistical analyses were performed using R studio version 1.1.453. One-way ANOVA, two-way ANOVA and Mixed-model ANOVA for repeated measures were used when appropriate. Pairwise comparisons were performed with Post hoc Bonferonni tests for multiple comparisons. Differences were considered statistically significant at *P values* < 0.05. Pearson correlation coefficients were performed using Excel (Microsoft Corporation) and significance of the correlations determined from the table of critical values.

## Results

### Energy intake, body weight, body composition and glucose homeostasis

**Figure 1:**
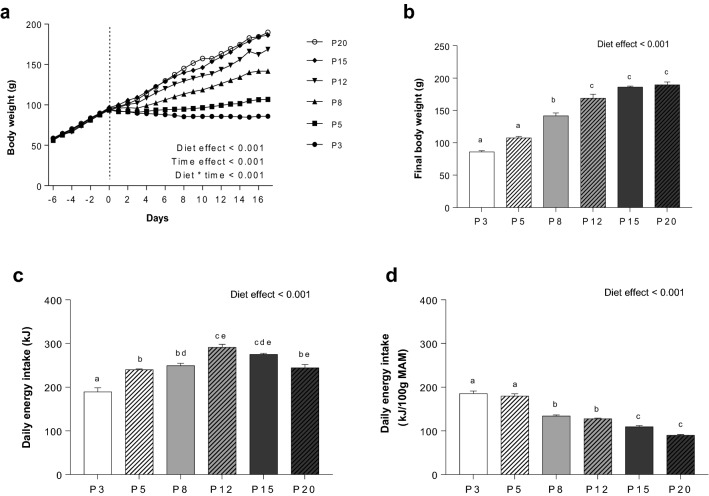
Effect of protein deficiency on body weight and energy intake. (**a**) Evolution of body weight; (**b**) Final body weight; (**c**) Daily energy intake; (**d**) Energy intake adjusted to 100 g MAM. Values are means ± SEM (n = 6 per group). ^a,b,c,d,e^Data that do not share the same letter are different at the *P* < 0.05 level.

Body weight gain was stopped or was very low when rats were fed the P3, P5 and P8 diets, but progressively recovered in those fed the P8 diet (Fig. 1a). Growth was unaffected in rats fed the P15 and P20 diets and slowed only slightly in those fed the P12 diet. At the end of the experimental period, rats fed the P3 and P5 diets had a body weight that was almost half of those fed the P15 and P20 diets (Fig. 1b). Mean rat daily energy intake was largest in P15 and P20-fed rats and lowest in those fed P3 and P5 diets (Fig. 1c; Table 1). When energy intake was adjusted to 100 g of MAM, energy intake increased quite linearly while dietary protein decreased from 20 to 3% (Fig. 1d; Table 1). The analysis of body composition revealed that lean mass content was lower in rats fed the P3, P5 and P8 diets, whereas no differences were observed between P12, P15 and P20-fed groups (Table 2). Fat mass was also low in P3 and P5-fed rats, but when fat mass was expressed relative to body weight, only the P8 group differed significantly from the P3 group.

**Table 1:** Absolute and relative values of energy intake.

	P3	P5	P8	P12	P15	P20	ANOVA
Cumulated food intake (g)	220.97 ± 11.18^a^	280.39 ± 2.34^b^	290.94 ± 6.76^bd^	340.04 ± 7.96^c^	320.94 ± 3.18^ cd^	285.03 ± 8.62^b^	< 0.001
Daily food intake (g/100 g MAM)	12.52 ± 0.63^a^	12.54 ± 0.51^a^	9.76 ± 0.14^bc^	9.14 ± 0.24^ cd^	7.99 ± 0.25^d^	6.66 ± 0.22^d^	< 0.001
Daily energy intake (kJ)	189.00 ± 9.56^a^	239.98 ± 2.01^b^	249.01 ± 5.79^bd^	291.23 ± 6.82^ce^	274.88 ± 2.72^cde^	244.29 ± 7.39^be^	< 0.001
Daily energy intake (kJ/100 g MAM)	185.06 ± 6.26^a^	179.65 ± 5.32^a^	133.78 ± 2.61^b^	127.42 ± 1.77^b^	109.24 ± 2.90^c^	89.84 ± 1.55^c^	< 0.001

Values are means ± SEM (n = 6 per group). ^a,b,c,d^Data that do not share the same letter are different at the *P* < 0.05 level.

**Table 2:** Absolute and relative values of body composition.

Diet	P3	P5	P8	P12	P15	P20	Test diet
Initial body weight (g)	58.61 ± 1.24	56.30 ± 0.98	58.90 ± 0.88	57.10 ± 1.64	58.26 ± 0.85	58.53 ± 0.99	NS
Final body weight (g)	85.98 ± 1.82^a^	106.7 ± 2.11^a^	141.82 ± 4.31^b^	168.8 ± 6.59^c^	186.1 ± 1.70^c^	189.67 ± 4.35^c^	< 0.001
MAM (g)	101.87 ± 1.87^a^	134.06 ± 3.41^b^	186.36 ± 4.94^c^	228.81 ± 6.54^d^	252.27 ± 5.20d^e^	272.07 ± 8.05^e^	< 0.001
Fat mass (g)	6.07 ± 0.42^a^	10.57 ± 0.64^ab^	16.33 ± 2.15^bc^	17.64 ± 1.75^c^	16.08 ± 0.66^bc^	17.66 ± 2.28^c^	< 0.001
Adiposity (Fat mass g/100 g body weight)	6.74 ± 0.46^a^	8.76 ± 0.45^ab^	9.54 ± 1.00^b^	8.51 ± 0.57^ab^	7.19 ± 0.27^ab^	7.20 ± 0.67^ab^	< 0.05
Lean body mass (g)	83.88 ± 1.55^a^	109.95 ± 2.81^b^	152.58 ± 3.87^c^	187.74 ± 5.21^d^	207.55 ± 4.31^e^	223.78 ± 6.33^e^	< 0.001
Liver (g)	3.35 ± 0.42^a^	3.95 ± 0.13^ab^	5.21 ± 0.30^b^	7.13 ± 0.29^c^	8.01 ± 0.39^ cd^	8.48 ± 0.43^d^	< 0.001
Kidney (g)	0.72 ± 0.03^a^	0.88 ± 0.03^a^	1.12 ± 0.04^b^	1.41 ± 0.03^c^	1.68 ± 0.05^d^	1.73 ± 0.04^d^	< 0.001
Gastrocnemius muscle	0.81 ± 0.01^a^	1.17 ± 0.01^b^	1.51 ± 0.03^c^	1.81 ± 0.04^d^	2.04 ± 0.06^de^	2.16 ± 0.09^e^	< 0.001
Carcass (g)	33.42 ± 0.77^a^	44.48 ± 1.00^b^	61.82 ± 0.94^c^	77.17 ± 1.70^d^	85.85 ± 1.76^e^	93.95 ± 2.80f.	< 0.001
Epididymal fat (g)	0.80 ± 0.09^a^	1.29 ± 0.08^a^	2.08 ± 0.15^b^	2.42 ± 0.23^b^	2.55 ± 0.09^b^	2.79 ± 0.29^b^	< 0.001
Mesenteric fat (g)	1.15 ± 0.12^a^	1.75 ± 0.14^ab^	2.45 ± 0.30^bc^	2.77 ± 0.32^bc^	2.89 ± 0.17^c^	3.15 ± 0.28^c^	< 0.001
Retroperitoneal fat (g)	0.84 ± 0.07^a^	1.51 ± 0.08^ab^	2.4 ± 0.31^bc^	2.98 ± 0.31^c^	2.95 ± 0.16^c^	3.30 ± 0.47^c^	< 0.001
Subcutaneous fat (g)	3.26 ± 0.28^a^	6.00 ± 0.47^ab^	9.39 ± 1.52^b^	9.45 ± 1.03^b^	7.68 ± 0.36^b^	8.40 ± 1.33^b^	< 0.01
Brown adipose tissue (g)	0.36 ± 0.01^a^	0.56 ± 0.02^b^	0.57 ± 0.03^b^	0.56 ± 0.03^b^	0.52 ± 0.02^b^	0.52 ± 0.04^b^	< 0.001

Values are means ± SEM (n = 6 per group). ^a,b,c,d^Data that do not share the same letter are different at the *P* < 0.05 level.

No major changes were observed for glucose homeostasis. Fast blood glucose did not differ between groups. Plasma insulin levels decreased in P5 rats, which differed from P15 rats, but not from the other groups or from the P20 control group.

### Total energy expenditure and motor activity

**Figure 2:**
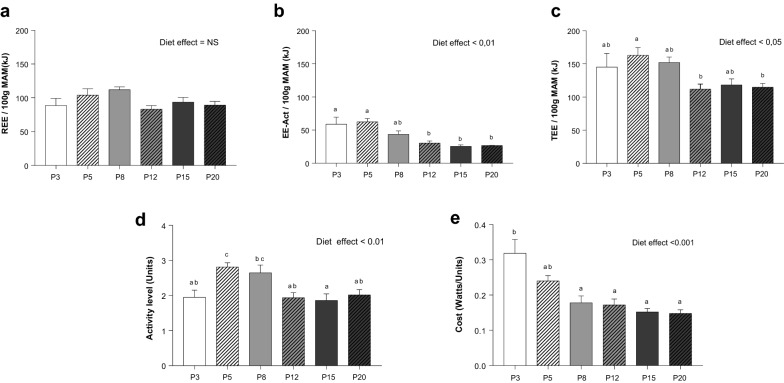
Component of energy expenditure in ad libitum-fed rats: resting energy expenditure (**a**: REE), energy expenditure of motor activity (**b**: EE—Act), total energy expenditure (**c**: TEE), activity level (**d**) and cost of activity (**e**). Values are means ± SEM (n = 6 per group). ^a,b,c,d^Data that do not share the same letter are different at the *P* < 0.05 level.

Energy expenditure was analyzed to understand why rats under severe protein-deficient diets (3% and 5%) ate more without gaining weight and fat mass (Table 2; Fig. 2). There was no significant effect of diet on REE (Fig. 2a), but there was a significant increase in EE-Act in rats fed the P3, P5 and P8 diets (Fig. 2b). As a result, TEE (REE + EE-Act) was increased from ~ 120 kJ in P12, P15 and P20-fed rats to 140–160 kJ in P3, P5 and P8-fed rats, the increase being significant only in those fed P5 (Fig. 2c). The increase in EE-Act was explained by an increase in activity level (Fig. 2d) and a 71% (NS) increase in activity cost in P5 rats. In comparison, P3-fed rats did not show an increased activity level although the cost of activity did increase 120% (Fig. 2e).

### Plasma FGF21 and expression of Fgf21 and other factors in liver, brown adipose tissue, duodenum and hypothalamus

**Figure 3:**
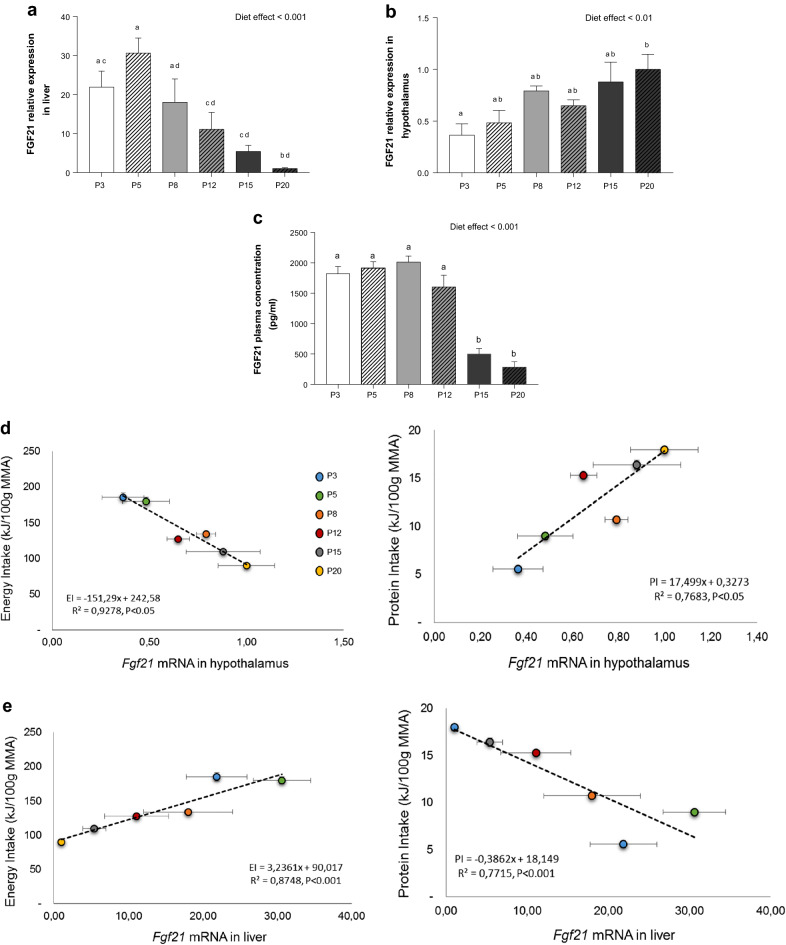
Expression of *Fgf21* mRNA in the liver (**a**) and the hypothalamus (**b**), and FGF21 plasma concentrations (**c**) two hours after ingestion of a 4 g test-meal. Correlations between mean group values of *Fgf21* mRNA hypothalamus values vs energy intake (EI) and protein intake (PI) (**d**), and correlation between *Fgf21* mRNA liver vs energy intake and protein intake (**e**). Values are means ± SEM (n = 6 per group). ^a,b,c,d^Data that do not share the same letter are different at the *P* < 0.05 level.

In the liver, *Fgf21* mRNA expression was increased by 20% in P3-fed rats and by 30% in P5-fed rats compared to those fed P20. *Fgf21* mRNA in P8, P12 and P15-fed rats was intermediate (Fig. 3a). Liver *Fgf21* mRNA correlated with plasma FGF21 levels was higher in P3, P5, P8 and P12 rats than in P15 and P20 rats (Fig. 3c). PGC-1α mRNA expression, an FGF21 regulator, was increased in P3 rats compared to P15 and P20 rats. The same results were found for KLB mRNA expression, demonstrating a difference between P3 and P12 rats. KLB mRNA encodes beta-Klotho, the co-receptor needed to activate the FGF21 receptor.

In brown adipose tissue, *Fgf21* mRNA was unaffected by the level of protein intake (Supplementary Table 3). In the hypothalamus, *Fgf21* mRNA expression decreased progressively from P20 to P3, but the decrease reached significance only when comparing P20-fed and P3-fed rats (Fig. 3b). The same result was found for *Fgf r2* mRNA expression, FGF21 receptor, whereas no effect was observed for the other isoforms (*Fgf r1 and Fgf r3)*.

Severe protein deficiency (3%) was associated with a decrease in *Pomc* and *Cartp* mRNA expression. No differences were observed for other neuropeptides involved in the control of food intake (*Npy*, *Mc4r, Npy2r, Agrp* genes) and in the duodenum, no changes were observed in the expression of cholecystokinin (Supplementary Table 3).

Moreover, liver *Fgf21* mRNA levels correlated strongly and positively with energy intake (R^2^ = 0.87, *P* < 0.001) and negatively with protein intake (R^2^ = 0.77, *P* < 0.001) (Fig. 3e). In contrast, hypothalamus *Fgf21* mRNA levels correlated negatively with energy intake (R^2^ = 0.92, *P* < 0.05) and positively with protein intake (R^2^ = 0.76, *P* < 0.05) (Fig. 3d).

### Expression of genes related to energy metabolism in the liver, adipose tissue and muscle

In order to investigate whether the increase of plasma FGF21 was related to energy expenditure markers, the present study also investigated the expression of genes involved in energy oxidation and browning of white adipose tissue, in brown and white adipose tissue. In brown adipose tissue, no effects were observed on gene expression of *Ucp1*, *Ucp2* and *Ucp3* (Supplementary Table 3). In white adipose tissue, no effects were observed on gene expression of *Ucp1* and *Ucp3,* suggesting that the increase of FGF21 did not stimulate the browning of white adipose tissue*.* There was a lower expression of *Ucp2* mRNA, which is present in most tissues, in P3 compared to P20-fed rats.

We also explored the effect of diet protein level on lipid oxidation markers in the liver and muscle and on lipogenesis in liver and white adipose tissue. In the liver, no changes were observed in mRNA-encoding CPT1, the rate-limiting protein for long-chain fatty acid entry into mitochondria and subsequent oxidation. In both liver and white adipose tissue, the low protein diets induced a stimulation of lipogenesis through the increase of ACC and FAS, which may be the consequence of the higher carbohydrate content of these diets. In muscle, no major changes were observed except a significantly higher ACCb mRNA abundance in P3 vs P20-fed rats without any changes in mRNA-encoding CPT1. This suggests reduced lipid oxidation due to a higher production of malonyl CoA.

## Discussion

This study aims to better understand the role played by FGF21 in metabolic and behavioral adaptations to protein restriction. To this end, young, growing rats were fed diets differing in their percentage of protein, including very low (3% and 5%), moderately low (8% and 12%), or adequate (15% and 20%) percentages^3^. A key result was that FGF21 expression inversely responded to protein intake in the liver and the hypothalamus, which explains the subsequent metabolic and behavioral adaptation to the level of protein intake.

Low protein diets have a major impact on the body weight gain of growing rats. The decrease in the diet’s protein content induced a decrease in body weight gain, mainly owing to the decrease in the gain of lean mass. In accordance with the literature, severely deficient diets (P3, P5 and P8) induced a large decrease in body weight gain compared to rats fed the P12, P15 and P20 diets^4,6,12^. Despite this decrease in body weight gain, we observed that rats under low protein diets have a higher energy intake relative to body size compared to rats fed P20. This is in line with several previous studies reporting that under protein restriction, growing rats increased their food intake or their relative food intake in order to meet protein requirements^5,6,24,38,39^. In comparison, other studies have contrastingly shown that protein restriction (4% and 5% casein) decreased food intake, though this effect could be related to the low palatability of the diet^40,41^. In our study, the increase in food intake under severely protein deficient diets (P3 and P5) was associated with a decrease in anorexigenic peptides, *Pomc* and *Cartp* mRNA, in the hypothalamus, as previously reported^42^.

Despite an increase in energy intake under a low protein diet, fat mass did not increase as much as expected. This limitation in fat mass gain was related to the concomitant increase in energy expenditure. It has been observed that a reduction of the dietary protein level is associated with an increase in energy expenditure^8,10–12,43^. In the present study, TEE was increased in P5-fed rats compared to those fed P12 and P20. In contrast, a very severe deficit in protein, at the level of 3%, seems too large to induce a significant increase in TEE compared to control rats under P20. The increase in TEE in P5 rats was mainly associated with the increase in the activity component of energy expenditure, and not with the REE. However, low protein diets (P3 and P5) did not induce hyperactivity, but did increase the cost of activity, allowing for a partial increase of energy expenditure regarding activity in the TEE. The same effect of an increased metabolic rate of motor activity associated with an increase in the cost of energy—and not with the level of activity—was previously observed in mice under a low protein diet^11^. This process allows the animals to increase their TEE, and therefore increase their energy intake without gaining fat mass. Metabolic analysis of pathways involved in energy expenditure confirmed that mRNAs encoding genes related to REE, such as fatty acid oxidation and thermogenesis (UCP), were not notably affected by low protein intake. In BAT, uncoupling protein (UCP1, UCP2 and UCP3) are regularly cited to play a role in thermogenesis. In addition, low protein diets impact their expression^44^. In this study, no significant effect of protein intake on UCP expression was observed in BAT, in accordance with different studies^12^. While other studies have shown that the increase in energy expenditure induced by low protein diets requires both UCP1 and FGF21^5,10,39,45–47^, results from the present study suggest that FGF21 does not require UCP1 to increase TEE^48,49^. We cannot exclude the possibility that animals may have developed FGF21 resistance under our experimental conditions. However, PGC1 alpha and KLB expression suggest that the liver is sensitive to circulating FGF21. These results must be confirmed by FGF21 signaling studies in both the hypothalamus and liver. Moreover, induction of liver-integrated stress response-driven nuclear protein 1 (NUPR1) is involved in FGF21 expression and secretion under low protein diets^49^. Thus, rather than increase energy expenditure through the activation of browning adipose tissue, FGF21 may more likely activate ketogenesis, as has been previously reported in transgenic mice with liver-specific overexpression of FGF21^16^.

In line with previous observations, liver *Fgf21* mRNA and FGF21 plasma concentration were increased in rats fed severely low protein diets^12,15,25^. In addition, the present results suggest that the threshold under which the liver responds to the low protein diet with an activation of FGF21 synthesis was below to P8 and for FGF21 secretion, was between P3 and P12. In other words, it is a value relatively higher than the threshold observed for liver mRNA expression. When recombinant FGF21 was injected in mice and monkeys, pharmacokinetic results showed that the half-life in plasma is short (0.5 to 2 h)^31^ and thus could not explain the difference of the threshold observed between liver mRNA and FGF21 plasma levels. However, the stability of endogenous glycosylated FGF21 is currently unknown, and the physiological half-life in circulation could be longer. In the present study, the decrease in a diet’s protein content was made at the expense of carbohydrates. However, it is unclear which of these two macronutrients is responsible for the increase in liver mRNA and plasma level of FGF21, and the consequential increase in energy intake and expenditure. Interestingly, a recent study by Zapata RC et al.^50^ using sympathetic system antagonists demonstrated that low protein diets with fixed carbohydrate content increased FGF21, promoted hyperphagia and sympathetically mediated an increase in energy expenditure. Indeed, these data confirmed that the low protein content of the diet, rather than a high carbohydrate content, was responsible for the increase of FGF21. In turn, it induced an increase in energy expenditure and food intake through a mechanism independent of browning adipose tissue, but through an increase of the energy cost of activity. How the sympathetic system is involved in this process remains to be determined, as does the nature of the relationship, if there is one, with FGF21 synthesis and secretion.

In addition, the present results show an inverse response of *Fgf21* expression to diet protein content in the liver and the hypothalamus. In contrast to the liver, *Fgf21* mRNA in the hypothalamus decreased in proportion to the decrease in protein content in the diet, with a progressive, fairly stable decrease from P20 to P3. The value of *Fgf21* mRNA in the hypothalamus was significantly lower in rats fed P3 vs P20. Of note, and in line with this result, it has been reported that tanycytes, which are located close to FGF21-sensitive neurons, produce and secrete FGF21^28^. Moreover, intracerebroventricular (ICV) injection of FGF21 markedly suppressed food intake in mice^51^, and ICV injection of FGF21 induced a shift in diet preferences to increase protein intake in mice adapted to a low-protein diet. Central FGF21 signaling was shown to be essential for these responses^23^. Furthermore, there is no effect of low-protein intake on orexigenic hypothalamic peptides AGRP and *NPY*. Therefore, the decrease of hypothalamic FGF21 could be involved in the higher food intake when the protein content of the diet is low. However, the effects of a low protein diet on energy expenditure^25,47,52^ and food intake^52^ were reversed in *Fgf21*-KO mice compared to Wild-type mice. These data showed that liver and circulating FGF21 are important in the adaptation to low protein intake but the role of the hypothalamic FGF21 gene expression needs to be also precisely identified and different hypotheses should be explored among the complex function previously associated to FGF21 in different tissues. It was shown that FGF21 is not only a hepatic signal, but it is also a signal secreted by different tissues such skeletal muscle in response to stress caused by a wide range of metabolic dysregulation and dysfunction. In this context, the liver-brain axis may act in synergy to prevent impaired tissue-specific function and damage in response to very low protein intake. Liver and thus circulating FGF21 induces an increase in energy expenditure that participates to an increase in appetite and food intake but may also be transferred across the blood brain barrier and directly act at the central level. Modulation of central hypothalamic FGF21 signaling may be so involved and it can be hypothesized that it is a combination of high circulating FGF21 and reduced expression of hypothalamic FGF21 that produce the association of increased appetite and preference for protein. Understanding the respective role of liver and hypothalamic FGF21 will require further experiments using specific KO mice and FGF21 antagonists.

This study confirms that reducing dietary protein to below the levels required to sustain optimal growth induced a large decrease in the gain of lean body mass and an increase in energy intake. Moreover, the present results show that protein deficiency induced an up-regulation of hepatic expression of FGF21 that increased TEE and induced a down-regulation of hypothalamic expression of FGF21 that could lead to the observed hyperphagia and preference for protein. The present study was performed in rat and the question of possible interspecies differences should be probably more carefully addressed. FGF21 expression in the hypothalamus has been reported both in rat and mice but whether the associated brain signaling pathways are strictly identical in these two species has not been precisely addressed. This question should be probably more carefully evaluated as some differences were previously observed for other mediators and hormones between different species. To our knowledge, our study is the first to describe the effect of protein deficiency on hypothalamic FGF21.

## Supplementary Information

Supplementary Tables.
