# Supplementary material for: Severe protein deficiency induces hepatic expression and systemic level of FGF21 but inhibits its hypothalamic expression in growing rats
Source: Sci Rep. 2021 Jun 14;11:12436. doi: 10.1038/s41598-021-91274-4 (PMC8203610; doi:10.1038/s41598-021-91274-4)
Supplement: Supplementary file 1 — Supplementary Tables. [file 41598_2021_91274_MOESM1_ESM.docx]

***Severe protein deficiency induces hepatic expression and systemic level of FGF21 but inhibits its hypothalamic expression in growing rats***

Joanna Moro^1^, Catherine Chaumontet^1^, Patrick C. Even^1^, Anne Blais^1^, Julien Piedcoq^1^, Claire Gaudichon^1^, Daniel Tomé^1^ and Dalila Azzout-Marniche^1^

^1^UMR PNCA, AgroParisTech, INRAe, Université Paris-Saclay, Paris, 75005, France

**Supplementary appendix:**

**Supplementary Table 1.** Macronutrient composition of diets. Diets were produced by the “Atelier de préparation des aliments”, UPAE, INRA, Jouy en Josas, France

|  | **P3** | **P5** | **P8** | **P12** | **P15** | **P20** |
| --- | --- | --- | --- | --- | --- | --- |
| **Weight content (g/kg)** |  |  |  |  |  |  |
| Milk proteins | 29 | 48 | 77 | 116 | 145 | 193.5 |
| Corn Starch | 717.9 | 701.5 | 676.6 | 643.1 | 618.1 | 576.4 |
| Sucrose | 115.8 | 113.2 | 109.1 | 103.6 | 99.6 | 92.8 |
| Soy Oil | 40 | 40 | 40 | 40 | 40 | 40 |
| Minerals | 35 | 35 | 35 | 35 | 35 | 35 |
| Vitamins | 10 | 10 | 10 | 10 | 10 | 10 |
| Cellulose | 50 | 50 | 50 | 50 | 50 | 50 |
| Choline | 2.3 | 2.3 | 2.3 | 2.3 | 2.3 | 2.3 |
| **Energy content (%)** |  |  |  |  |  |  |
| Protein | 3 | 5 | 8 | 12 | 15 | 20 |
| Carbohydrate | 86.6 | 84.6 | 81.6 | 77.6 | 74.6 | 69.5 |
| Fat | 9.3 | 9.3 | 9.3 | 9.3 | 9.3 | 9.3 |
| Energy density (kJ/g) | 14.54 | 14.55 | 14.55 | 14.56 | 14.56 | 14.57 |

**Supplementary Table 2.** Primer sequences used for liver, brown adipose tissue, epididymal adipose tissue, muscle and hypothalamus mRNA analysis

| Proteine | GENES | Full-length name | Forward primer (5’ to 3’) | Reverse Primer (3’ to 5’) |
| --- | --- | --- | --- | --- |
| - | *18S* | ribosomal RNA | ACGGAAGGGCACCACCAGGAG | GCACCACCACCCACGGAAACG |
| ACCa | *Acaca* | acetyl-CoA carboxylase a | TGGTGCAGAGGTACCGAAGTG | CGTAGTGGCCGTTCTGAAACT |
| ACCb | *Acacb* | acetyl-CoA carboxylase b | GGGCTCCCTGGATGACAAC | GCTCTTCCGGGAGGAGTTCT |
| AGRP | *Agrp* | agouti related neuropeptide | GTTCCCAGAGTTCCCAGGTCTAA | GTGTCTCAGGTCTGCAGTTA |
| CART | *Cartp* | cocaine- and amphetamine-regulated transcript | CCGAGCCCTGGACATCTACTC | AAATACTGACCAGCTCCTTCTCATG |
| CCK | *Cck* | cholecystokinin | CAGGTCCGCAAAGCTCCTT | TCCAGGCTCTGCAGGTTCT |
| CD36 | *Cd36* | cluster of differenciation 36 | CATGATTAATGGCACAGATGCA | GCAAATGTCAGAGGAAAAGAACCT |
| CPT1a | *Cpt1a* | carnitine palmitoyl transferase 1a | TCTCTGGATGCGGTAGAAAAGG | CTCTATATCCCTGTTCCGATTCGT |
| CPT1b | *Cpt1b* | carnitine palmitoyl transferase 1b | CAGCCATGCCACCAAGATC | CTTGGGCAGTGATGTTTGGA |
| FAS | *Fasn* | fatty acid synthase | TGCTCCCAGCTGCAGGC | GCCCGGTAGCTCTGGGTGTA |
| FGF21 | *Fgf21* | fibroblast growth factor 21 | CCTGGAGCTCAAAGCCTTGA | AAACTGCAGGCCTCAGGATC |
| FGF R1 | *Fgfr1* | fibroblast growth factor receptor 1 | TGGCACCTGAGGCATTGTT | CCCCAAAAGACCACACATCAC |
| FGF R2 | *Fgfr2* | fibroblast growth factor receptor 2 | CCCAAAAATGTATCCATCGAGACT | TCATCACGTTGTTTTCTGTTACCA |
| FGF R3 | *Fgfr3* | fibroblast growth factor receptor 3 | GCCTGCTGACCCCAAGTG | CCTGTCCAAAGCAGCCTTCT |
| KLB | *KLB* | beta-Klotho | CCAGGAGAGAAGGGAAATTTAACA | TCGAATGCGCCTTGATCA |
| MC4R | *Mc4r* | melanocortin 4 receptor | CCGAACCCAGAAGAGACCAA | CTAGGAGCAGGGTCAGAAGCA |
| NPY | *Npy* | neuropeptide Y | GGGAGCCTGAGAAACGGC | CCTGGTGGTGGCATGCAT |
| Y2R | *Npy2r* | neuropeptide Y receptor Y2 | CCGCTCCTGCTTCTGACTC | ACCCAAAGCAGGTCCGATT |
| PGC-1α | *Ppargc1a* | PPARG coactivator 1 alpha | CAAGGTCCCCAGGCAGTAGA | GGTGTCTGTAGTGGCTTGATTCAT |
| POMC | *Pomc* | proopiomelanocortin | AGGCCTTTCCCCTAGAGTTCAA | GTCGGCCTTCTCGGTATCC |
| UCP1 | *Ucp1* | mitochondrial uncoupling protein 1 | CGTACCAAGCTGTGCGATGT | GACCCGAGTCGCAGAAAAGA |
| UCP2 | *Ucp2* | mitochondrial uncoupling protein 2 | TGAAAGCCAACCTCATGACAGA | CAATGACGGTGGTGCAGAAG |
| UCP3 | *Ucp3* | mitochondrial uncoupling protein 3 | GGGATCCTGGAACGTGATGA | TGGAGATTCCCGCAGTACCT |

**Supplementary Table 3.** mRNA abundance in liver, brown adipose tissue, epididymal adipose tissue, muscle and hypothalamus of growing rats fed with 3, 5, 8, 12, 15 or 20% protein in diet two hours after meal onset. Values are means ± SEM (n=6 per group). ^a, b, c, d^ Data that do not share the same letter are different at the p<0.05 level

| Diet |  | P3 | P5 | P8 | P12 | P15 | P20 | Test diet |
| --- | --- | --- | --- | --- | --- | --- | --- | --- |
| Liver | FGF21 | 21.89 ± 4.10^ac^ | 30.63 ± 3.86^a^ | 18.02 ± 5.97^ad^ | 11.09 ± 4.33^cd^ | 5.41 ± 1.56^cd^ | 1.00 ± 0.22^bd^ | <0.001 |
|  | FGF R1 | 0.50 ± 0.17 | 0.97 ± 0.04 | 0.93 ± 0.14 | 0.77 ± 0.06 | 0.87 ± 0.09 | 1.00 ± 0.17 | NS |
|  | ACCa | 2.69 ± 0.19^a^ | 1.82 ± 0.11^bc^ | 2.35 ± 0.25^ac^ | 1.17 ± 0.20^b^ | 1.14 ± 0.21^b^ | 1.00 ± 0.11^b^ | <0.001 |
|  | FAS | 3.06 ± 0.28^a^ | 2.08 ± 0.30^ab^ | 2.71 ± 0.38^a^ | 1.05 ± 0.24^b^ | 1.26 ± 0.20^b^ | 1.00 ±0.26^b^ | <0.001 |
|  | CPT1a | 2.41 ± 0.37 | 2.56 ± 0.32 | 1.73 ± 0.31 | 1.32 ± 0.15 | 1.13 ± 0.10 | 1.00 ± 0.17 | NS |
|  | MTTP | 1.75 ± 0.17 | 1.52 ± 0.23 | 1.56 ± 0.17 | 1.09 ± 0.18 | 1.12 ± 0.16 | 1.00 ± 0.15 | <0.05 |
|  | PGC1α | 3.32 ± 0.68^a^ | 1.58 ± 0.41^ab^ | 2.65 ± 0.32^ab^ | 1.67 ± 0.31^ab^ | 0.96 ± 0.31^b^ | 1.00 ± 0.14^b^ | <0.01 |
|  | KLB | 2.52 ± 0.41^a^ | 1.49 ± 0.20^ab^ | 1.63 ± 0.26^ab^ | 1.05 ± 0.17^b^ | 0.92 ± 0.19^b^ | 1.00 ± 0.15^b^ | <0.001 |
| Brown adipose tissue | FGF21 | 1.27 ± 0.33 | 1.71 ± 0.22 | 1.56 ± 0.31 | 1 ± 0.10 | 1.07 ± 0.16 | 1.00 ± 0.17 | NS |
|  | UCP1 | 1.18 ± 0.35 | 1.71 ± 0.16 | 1.22 ± 0.14 | 1.28 ± 0.35 | 0.92 ± 0.16 | 1.00 ± 0.20 | NS |
|  | UCP2 | 0.84 ± 0.14 | 1.33 ± 0.21 | 1.48 ± 0.29 | 1.66 ± 0.30 | 1.24 ± 0.26 | 1.00 ± 0.21 | NS |
|  | UCP3 | 0.83 ± 0.10 | 1.24 ± 0.16 | 1.11 ± 0.14 | 0.83 ± 0.12 | 0.95 ± 0.13 | 1.00 ± 0.14 | NS |
| Epididymal adipose tissue | ACCb | 1.50 ± 0.06^a^ | 1.46 ± 0.05^ab^ | 1.24 ± 0.13^ac^ | 1.20 ± 0.09^ac^ | 1.02 ± 0.11^bc^ | 1.00 ± 0.14^c^ | <0.01 |
|  | FAS | 1.68 ± 0.07^a^ | 1.71 ± 0.08^a^ | 1.48 ± 0.14^ab^ | 1.35 ± 0.20^ab^ | 1.20 ± 0.20^ab^ | 1.00 ± 0.13^b^ | <0.05 |
|  | CPT1b | 0.76 ± 0.09 | 0.84 ± 0.10 | 1.08 ± 0.29 | 0.75 ± 0.11 | 0.94 ± 0.09 | 1.00 ± 0.16 | NS |
|  | CD36 | 1.40 ± 0.14 | 1.37 ± 0.12 | 1.25 ± 0.10 | 1.19 ± 0.17 | 1.03 ± 0.08 | 1.00 ± 0.15 | NS |
|  | UCP1 | 58.90 ± 40.41 | 13.88 ± 6.56 | 6.79 ± 1.14 | 5.45 ± 1.65 | 2.18 ± 0.63 | 1.00 ± 0.21 | NS |
|  | UCP2 | 0.59 ± 0.06^a^ | 0.77 ± 0.06^ab^ | 0.80 ± 0.08^ab^ | 0.83 ± 0.09^ab^ | 0.97 ± 0.02^ab^ | 1.00 ± 0.15^b^ | <0.05 |
|  | UCP3 | 1.07 ± 0.13 | 0.89 ± 0.08 | 0.99 ± 0.17 | 0.96 ± 0.14 | 0.74 ± 0.10 | 1.00 ± 0.14 | NS |
| Muscle | ACCb | 2.49 ± 0.34^a^ | 2.00 ± 0.53^ab^ | 1.28 ± 0.22^ab^ | 1.23 ± 0.21^ab^ | 1.26 ± 0.11^ab^ | 1.00 ± 0.07^b^ | <0.01 |
|  | FAS | 9.55 ± 4.13 | 10.35 ± 6.07 | 3.49 ± 1.44 | 7.12 ± 1.57 | 2.06 ± 0.47 | 1.00 ± 0.15 | NS |
|  | CPT1b | 1.34 ± 0.14 | 1.12 ± 0.10 | 0.96 ± 0.04 | 1.08 ± 0.08 | 1.23 ± 0.08 | 1.00 ± 0.06 | NS |
|  | CD36 | 1.06 ± 0.09 | 1.02 ± 0.15 | 0.92 ± 0.07 | 1.14 ± 0.24 | 1 ± 0.10 | 1.00 ± 0.07 | NS |
|  | FGF21 | 0.36 ± 0.11^a^ | 0.48 ± 0.12^ab^ | 0.79 ± 0.05^ab^ | 0.65 ± 0.06^ab^ | 0.88 ± 0.19^ab^ | 1.00 ± 0.15^b^ | <0.01 |
| Hypothalamus | FGF R1 | 0.48 ± 0.08 | 0.63 ± 0.10 | 0.85 ± 0.13 | 0.78 ± 0.08 | 0.96 ± 0.22 | 1.00 ± 0.16 | NS |
|  | FGF R2c | 0.51 ± 0.05 | 0.63 ± 0.08 | 0.87 ± 0.12 | 0.84 ± 0.04 | 0.91 ± 0.21 | 1.00 ± 0.11 | <0.05 |
|  | FGF R3 | 0.63 ± 0.09 | 0.73 ± 0.13 | 0.98 ± 0.13 | 1 ± 0.05 | 0.87 ± 0.13 | 1.00 ± 0.15 | NS |
|  | AGRP | 0.41 ± 0.08 | 0.63 ± 0.14 | 0.77 ± 0.11 | 0.59 ± 0.08 | 0.83 ± 0.14 | 1.00 ± 0.11 | <0.05 |
|  | MC4R | 0.67 ± 0.06 | 0.89 ± 0.08 | 0.87 ± 0.10 | 0.79 ± 0.07 | 0.89 ± 0.14 | 1.00 ± 0.10 | NS |
|  | NPY | 1.34 ± 0.12 | 1.30 ± 0.16 | 1.10 ± 0.23 | 0.97 ± 0.13 | 1.11 ± 0.31 | 1.00 ± 0.13 | NS |
|  | NPY2r | 0.54 ± 0.09 | 0.63 ± 0.13 | 0.85 ± 0.09 | 0.73 ± 0.06 | 0.82 ± 0.11 | 1.00 ± 0.14 | NS |
|  | CART | 0.33 ± 0.12^a^ | 0.60 ± 0.18^ab^ | 0.91 ± 0.11^ab^ | 0.80 ± 0.12^ab^ | 0.82 ± 0.05^ab^ | 1.00 ± 0.15^b^ | <0.05 |
|  | POMC | 0.19 ± 0.07^a^ | 0.65 ± 0.24^ab^ | 0.98 ± 0.22^b^ | 0.80 ± 0.08^ab^ | 0.95 ± 0.14^ab^ | 1.00 ± 0.21^b^ | <0.05 |
|  | PGC-1α | 0.85 ± 0.13^a^ | 0.88 ± 0.11^ab^ | 0.93 ± 0.14^ab^ | 0.54 ± 0.07^ab^ | 0.88 ± 0.12^b^ | 1.00 ± 0.21^b^ | <0.01 |
| Duodenum | CCK | 0.98 ± 0.06 | 0.89 ± 0.10 | 1.05 ± 0.10 | 0.83 ± 0.09 | 0.94 ± 0.13 | 1.00 ± 0.07 | NS |

**Supplementary Table 4.** Glycaemia and insulin levels in growing rats fed with 3, 5, 8, 12, 15 or 20% protein in diet two hours after meal onset. Values are means ± SEM (n=6 per group). ^a, b, c, d^ Data that do not share the same letter are different at the p<0.05 level

|  | P3 | P5 | P8 | P12 | P15 | P20 | Test diet |
| --- | --- | --- | --- | --- | --- | --- | --- |
| Glycaemia | 252 ± 17.83 | 253 ±26.97 | 272 ± 25.42 | 252 ± 18.93 | 228 ±10.64 | 232.67 ± 9.08 | NS |
| Insulin | 803.02 ±197.17^ab^ | 639.51 ± 69.77^a^ | 790.77 ±104.02^ab^ | 1047.40 ± 173.72^ab^ | 1291.99 ± 157.92^b^ | 1087.66 ± 123.11^ab^ | <0.01 |
